# Inactivation of ZSCAN18 by promoter hypermethylation drives the proliferation via attenuating TP53INP2-mediated autophagy in gastric cancer cells

**DOI:** 10.1186/s13148-023-01425-9

**Published:** 2023-01-17

**Authors:** Bin Li, Baoqing Ren, Gang Ma, Fenglin Cai, Pengliang Wang, Yi Zeng, Yong Liu, Li Zhang, Yang Yang, Han Liang, Rupeng Zhang, Jingyu Deng

**Affiliations:** 1 Department of Gastric Surgery, Tianjin Medical University Cancer Institute & Hospital, National Clinical Research Center for Cancer, Key Laboratory of Cancer Prevention and Therapy, Tianjin, Tianjin’s Clinical Research Center for Cancer, Tianjin, 300060 People’s Republic of China; 2 Department of Gastroenterology and Pancreatic Surgery, ShanXi Provincial People’s Hospital, Taiyuan, 030000 People’s Republic of China

**Keywords:** Gastric cancer, ZSCAN18, Methylation, Carcinogenesis, Autophagy

## Abstract

**Background:**

Zinc finger and scan domain containing 18 (ZSCAN18) belongs to the zinc finger transcription factor superfamily, which consists of hundreds of members that play critical roles in all steps of tumorigenesis.

**Methods:**

This study aims to investigate the roles of ZSCAN18 in gastric cancer (GC). The expression level in GC and the clinicopathologic features of ZSCAN18 were detected by immunohistochemistry staining. Methylation of ZSCAN18 promoter in GC tissues and cell lines was analyzed via MassARRAY; the same method was used to detect GC cell lines demethylated by 5-aza-2′-deoxycytidine treatment. The biological function of ZSCAN18 in GC cells was verified by in vitro and in vivo experiments. The downstream molecular mechanism of ZSCAN18 was explored using RNA next-generation sequencing, immunofluorescence and chromatin immunoprecipitation.

**Results:**

Our work revealed ZSCAN18 expression was markedly reduced in GC tissues compared with adjacent normal tissues as a result of hypermethylation in GC. Likewise, ZSCAN18 expression was significantly reduced in a panel of GC cell lines as a result of the densely methylated ZSCAN18 promoter. Functionally, ZSCAN18 overexpression inhibited the biological progression of GC cells, which was characterized by weaken proliferation, enhanced autophagy and suppressed tumor growth. ZSCAN18 acted as a transcription factor and played an important role in binding to the promoter of tumor protein 53-induced nuclear protein 2 (TP53INP2), and we also confirmed the anti-tumor effect of TP53INP2 in GC. Furthermore, the knockdown of TP53INP2 alleviated the inhibiting effects of ZSCAN18 in GC cells by in vitro and in vivo experiments.

**Conclusions:**

Collectively, this study unveiled that ZSCAN18 played an anticancer role in GC by promoting autophagy and transcriptional regulation of TP53INP2 and provided a promising target for the diagnosis and treatment of GC.

## Background

Gastric cancer (GC), as a complex heterogeneous disease, is the fifth most common cancer and the third most common cause of cancer deaths globally [1–3]. Although diagnostic and therapeutic strategies for advanced GC have improved significantly in recent years, the treatment remains unsatisfactory, and the prognosis remains poor. Therefore, the molecular mechanisms of GC pathogenesis should be elucidated to explore potential therapeutic targets for clinical use.

Zinc finger (ZNF) transcription factors (TF) represent the largest TF superfamily, and its members play critical roles in all aspects of cellular processes. Zinc finger and scan domain containing 18 (ZSCAN18) is a ZSCAN TF (a subfamily of ZNF TFs). ZSCAN18 is also known as ZNF447 and is located in chromosome 19q13.43. Recently, hypermethylation in the promoter of ZSCAN18 has been identified in various types of cancers, such as esophageal cancer [4], renal cell carcinoma [5], cholangiocarcinoma [6, 7], colorectal cancer [8, 9], GC [9–11], hepatocellular carcinoma [11], and pancreatic cancer [9–11]. Low protein expression of ZSCAN18 is reported in these malignancies, especially in gastrointestinal cancers. These findings strongly suggest that ZSCAN18 exerts anti-tumor effects. However, the mechanisms and functions of hypermethylated ZSCAN18 in GC remain poorly understood.

In this study, we analyzed the epigenetic regulation and functions of ZSCAN18 and revealed that promoter hypermethylation contributed to low ZSCAN18 expression in GC. We showed that ZSCAN18 hypermethylation in GC was correlated with prognostic outcomes. We also confirmed that ZSCAN18 high expression inhibited the proliferation of GC cells, both in vitro and in vivo. Our further investigation revealed that ZSCAN18 could bind to tumor protein 53-induced nuclear protein 2 (TP53INP2) promoter and promote autophagy, which might be related to the inhibitory effect of ZSCAN18 on cell proliferation. Most importantly, the knockdown of TP53INP2 alleviated the inhibiting effects of ZSCAN18 overexpression in GC cells. These results indicated that ZSCAN18 induced autophagy and suppressed GC proliferation through transcriptional regulation of TP53INP2. Moreover, ZSCAN18 expression may be involved in GC with a potential therapeutic target value.

## Materials and methods

### Data availability

The mRNA expression data and methylation data were obtained from the Gene Expression Omnibus (GEO) database with the login numbers GSE33335 and GSE30601.

### Tissue sample collection and follow-up

The tissues used for immunohistochemistry (IHC) were composed of 83 pairs of tumor and adjacent non-tumor tissues that were collected from patients with GC who underwent curative gastrectomy at Tianjin Medical University Cancer Hospital (Tianjin, China) between January 2004 and August 2007. All patients underwent standard follow-up after curative surgery. The median follow-up time was 22 months (range 3–70 months). The tissue used for the MassARRAY methylation test consisted of 97 cancerous tissues and 6 paracancer tissues from patients undergoing radical gastrectomy between January 2003 and July 2007 at Tianjin Medical University Cancer Hospital (Tianjin, China), of which 97 cancerous tissues were closely observed. The median follow-up time was 17 months (range 2–85 months). The clinical data included the patient’s age, sex, endoscopy result, chest X-ray, B-mode ultrasound and surgical methods. Between August and November 2019, we collected 30 pairs of GC and adjacent non-tumor tissues for RNA extraction. The study was conducted with patient consent. The experimental study protocol and the use of clinical data were approved by the Ethics Committee of the Cancer Institute of Tianjin Medical University and the Cancer Hospital of Tianjin Medical University (Tianjin).

### Cell culture and transfection

The cell lines of MKN45, NCI-N87, BGC-823, MGC-803, SGC-7901, SNU-1, KATO-III and GES-1 were cultured in RPMI 1640 medium (Gibco, Carlsbad, CA, USA) supplemented with 10% fetal bovine serum (FBS, HyClone) and 1% antibiotics (penicillin/streptomycin) (Gibco). AGS cells were cultured in F12k (Gibco) with 10% FBS and 1% antibiotics. The HEK293T cell line was cultured in Dulbecco’s modified Eagle medium (DMEM) (Gibco) with 10% FBS and 1% antibiotics. Cells were maintained at 37 °C with a 5% CO_2_ atmosphere. The expression levels of ZSCAN18 and TP53INP2 were enhanced by plasmids (plVX-IRES-Puro-ZSCAN18) and (plVX-IRES-Puro-TP53INP2), respectively, and an empty vector was transfected into the cells (pLVX-IRES-Puro-vector) as control. The shRNA against TP53INP2 interference sequence was synthesized (shTP53INP2, 5′-TGGACGGCTGGCTCATC-3). Western blot and quantitative real-time polymerase chain reaction (qRT-PCR) were used to verify the transfection efficiency.

### Immunohistochemistry

Further 83 paired GC and matched adjacent non-tumor tissues were retrieved from Tianjin Medical University Cancer Hospital (Tianjin, China), Xijing Hospital of Air Force Medical University (Xi’an, China) and Renji Hospital of Shanghai Jiao Tong University School of Medicine (Shanghai, China) between August 2004 and December 2007, and sent to Shanghai Outdo Biotech Company (Shanghai, China) for tissue microarray analysis to evaluate the expression of ZSCAN18. The antibody concentrations of ZSCAN18 and TP53INP2 were 1:150 and 1:300, respectively. The staining intensity of ZSCAN18 and TP53INP2 was estimated by three independent pathologists without knowledge of the clinical data. The staining intensity was divided into 0 (negative), 1 (weak), 2 (medium), and 3 (strong) ranks. Zero and 1 were considered negative; 2 and 3, positive.

### Western blot

The western blot experiment was conducted in accordance with standard procedures. The primary antibodies used for this research were as follows: anti-ZSCAN18 (TA505326, OriGene), anti-VINCULIN (ab219649, Abcam), anti-p62/SQSTM1 (#16177S, CST), anti-LC3A/B (#12741S, CST) and anti-TP53INP2 (ab273012, Abcam).

### Cell viability assay

Cell viability was detected using a Cell Counting Kit 8 (CCK-8) reagent. After the cell count, cells were placed in a 96-well culture plate; the AGS cell line had 1 × 10^3^ cells per well, and the NCI-N87 cell line had 3 × 10^3^ cells per well. Testing started 2 h after the addition of 10 µl of the CCK-8 reagent. AGS was tested once a day, and NCI-N87 was tested once every other day. Another way to assess cell viability was to measure adenosine triphosphate (ATP) levels using the Cell Titer-Glo assay (Promega, Cat#G7570). Chloroquine (CQ; HY-17589A) and rapamycin (RAP; HY-10219) were purchased from MedChemExpress (MCE, USA) and were treated at a concentration of 10 μM (CQ) and 100 nM (RAP) for 24 h, respectively. The ZSCAN18-overexpressed AGS, ZSCAN18-overexpressed NCI-N87 and control cells were cultured in 96-well plates with or without CQ/RAP.

### Colony formation assay

The colony-forming method was used to detect the proliferation of GC cells. We conducted experiments on AGS and NCI-N87 cell lines using 1000 AGS cells per well and 8000 NCI-N87 cells per well. After the cell count, the cells were inoculated into 6-well plates and incubated at 37 °C for 12 to 14 days. Cell fluid was changed periodically until a visible clone formed.

### Animal experiment

Ten 4-week-old female BALB/C nude mice (Vital River) were purchased to construct a subcutaneous tumor xenograft. A total of 3 × 10^6^ cells were injected into the lateral dorsal subcutaneous flank of nude mice. Xenograft tumor formation was measured every 4 days. Three weeks after injection, the nude mice were euthanized by cervical dislocation killing after intraperitoneal injection of 2% pentobarbital sodium (0.5 mL), and the tumor volume (*V*) was measured by the formula: *V* = 1/2 × length × (width)^2^. Tumor tissues were excised for immunohistochemistry.

### 5-Aza-2′-deoxycytidine treatment

AGS and NCI-N87 cells were laid 12 h in advance, and the cell density was approximately 30% on the next day. Cells were incubated with a concentration of 2 μM of 5-aza-2′-deoxycytidine (DAC, Sigma, St. Louis, MO). The liquid was changed every 24 h. The treatment lasted 96 h.

### RNA sequencing and analysis

NCI-N87 cells were stably transfected with PLVX-IRES-Puro-ZSCAN18 or empty vector (PLVX-IRES-Puro). RNA from total samples was isolated and purified using TRIzol (Invitrogen, CA, USA). Then, NanoDrop ND-1000 (NanoDrop, Wilmington, DE, USA) was used to control the quantity and purity of total RNA. The captured mRNA was fragmented at 94 °C for 5 to 7 min using a magnesium ion fragmentation kit (NEBNext^®^ RNA Fragmentation Module, article no. E6150S, USA). cDNA was synthesized from the segmented RNA using Invitrogen SuperScript™ II Reverse Transcriptase (article no. 1896649, CA, USA). Illumina NovaSeq™ 6000 (LC Bio Technology Co., Ltd., Hangzhou, China) was used for double-terminal sequencing according to the standard operation, and the sequencing mode was PE150. Cutadapt (https://cutadapt.readthedocs.io/en/stable, version for Cutadapt 1.9) was used to remove the joint plane raw data processing software. A significant difference was analyzed between samples, and the multiple of the difference (fold change [FC] > 2 or [FC] < 0.5 with a *p* value < 0.05) defined differentially expressed genes (DEGs), which were analyzed for Kyoto Encyclopedia of Genes and Genomes (KEGG) enrichment using R packs.

### MassARRAY analysis of the methylation level of ZSCAN18

Instructions to extract cells or tissues using the DNA extraction kit (BioTeKe Corporation) were observed. The DNA samples to be tested were processed using a commercial NaHSO_3_ kit (ZYMO). The gene fragments to be detected were enriched and amplified by PCR reaction, and the product length was 200 to 700 bp. The PCR product was treated with shrimp alkaline phosphatase to remove free deoxyribonucleoside-5′-triphosphates (dNTPs) in the system. A transcriptase digestion reaction was followed by resin purification. The purified products of the resin were transported to a 384-well SpectroCHIP® bioarray (Agena, Inc.) using an Agena NanodispenserRS1000 dot sampling instrument (Agena, Inc.). EpiTYPER™ software provided advanced and convenient quantitative analysis of DNA methylation.

### Binding motif prediction

The chromatin immunoprecipitation next-generation sequencing (ChIP-seq) information of ZSCAN18 was acquired from the Cistrome Data Browser database (http://cistrome.org/db/#) and then visualized using the UCSC browser. The predicted binding sequences at −100 bp to +2000 bp from the reference sequence (RefSeq) transcription start site were extracted from the U.S. National Library of Medicine (https://www.ncbi.nlm.nih.gov) and were then inputted into the ChIP-seq database of ZSCAN18. The DNA sequences were extracted from an obviously higher peak, and primers were designed in sections for the chromatin immunoprecipitation (ChIP) experiment.

### ChIP experiments followed previous described protocol

The ChIP assay was performed using the ChIP-IT^®^ Express Enzymatic Magnetic Chromatin Immunoprecipitation Kit & Enzymatic Shearing Kit (Cat No. 53009 & 53035, Active Motif, USA) according to the manufacturer's instructions. Briefly, stable ZSCAN18-expressing AGS or NCI-N87 cells and vector-expressing cells were cross-linked with formaldehyde, which were collected in centrifuge tubes for protein extraction. The chromatin was sheared to 200–1000 bp by enzymatic shearing from extracted cross-linked nuclei. The sheared chromatin was incubated with an antibody of ZSCAN18 (UM500081, OriGene); the antibody-bound DNA complexes were precipitated through the use of magnetic Protein G-coupled beads. The same amount of non-specific immunoglobulin G (IgG) is used as control. The captured chromatin was then eluted, the cross-links were reversed, and the recovered DNA was analyzed by qRT-PCR using the primer of TP53INP2 (5′-GGTGGGAAAGCAGAGTGTGT-3).

### Fluorescence microscope

The GC cells were transfected with recombinant mRFP‐GFP‐LC3 adenovirus. In green- and red‐merged pictures, autophagosomes were shown as yellow puncta, while autolysosomes were shown as red puncta. Otherwise, autophagic flux was detected using fluorescence microscope (Zeiss).

### Statistical analysis

IBM SPSS Statistics (version 24.0; Armonk, NY, USA) and GraphPad Prism 8 software (GraphPad Software Inc., La Jolla, CA, USA) were used to analyze the data for this study. The t test was used to compare statistical differences between the two groups. The chi-squared test and Fisher’s exact test were used to analyze the relationship between methylation and various clinicopathological parameters. Overall survival was determined by log-rank test and the Kaplan–Meier method. The Spearman correlation test was used to analyze the correlation between gene expression and the methylation level. A receiver operating characteristic (ROC) curve was used to evaluate the diagnostic value of methylation sites in cancerous and adjacent tissues. In univariate analysis, survival differences were estimated using the Kaplan–Meier method (log-rank test), and independent prognostic factors were subsequently identified in Cox proportional risk regression models for multivariate analysis. *p* < 0.05 was considered statistically significant.

## Results

### Downregulation of ZSCAN18 in primary GC is associated with poor survival

**Fig. 1:**
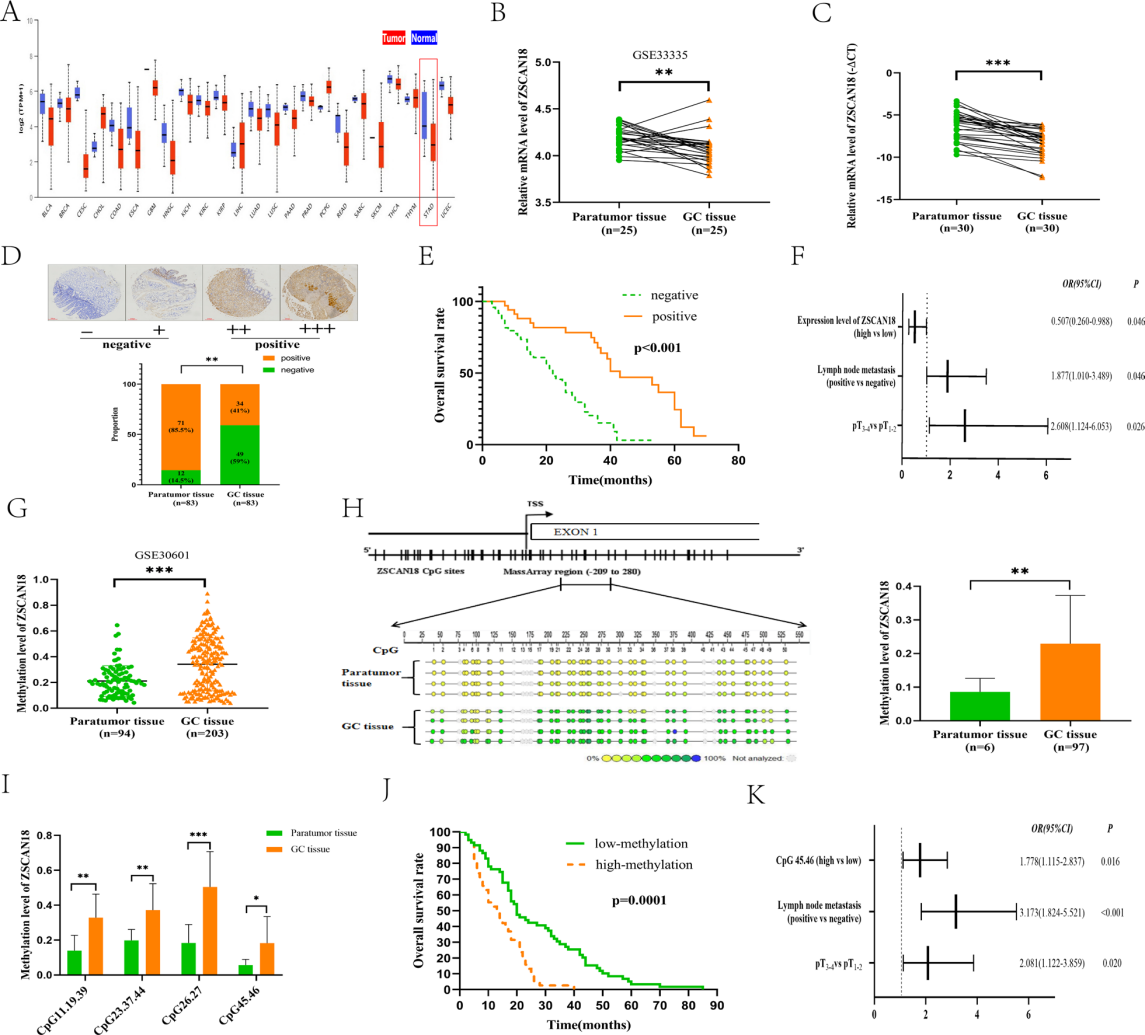
Deregulation of ZSCAN18 expression in GC is correlated with poor prognosis and high methylation. **A** Bioinformatics analysis of TCGA database (http://ualcan.path.uab.edu) between tumor tissues (red) and normal tissues (blue) in various cancers. BLCA: bladder urothelial carcinoma; BRCA: breast invasive carcinoma; CESC: cervical squamous cell carcinoma and endocervical adenocarcinoma; CHOL: cholangiocarcinoma; COAD: colon adenocarcinoma; ESCA: esophageal carcinoma; GBM: glioblastoma multiforme; HNSC: head and neck squamous cell carcinoma; KICH: kidney chromophobe; KIRC: kidney renal clear cell carcinoma; KIRP: kidney renal papillary cell carcinoma; LIHC: liver hepatocellular carcinoma; LUAD: lung adenocarcinoma; LUSC: lung squamous cell carcinoma; PAAD: pancreatic adenocarcinoma; PRAD: prostate adenocarcinoma; PCPG: prostate adenocarcinoma; READ: rectum adenocarcinoma; SARC: sarcoma; SKCM: skin cutaneous melanoma; THCA: thyroid carcinoma; THYM: thymoma; STAD: stomach adenocarcinoma; UCEC: uterine corpus endometrial carcinoma. **B** Bioinformatics analysis from the public datasets (GSE33335) in adjacent non-tumor tissues and tumor tissues. **C** mRNA expression levels of ZSCAN18 in GC tissue and matched adjacent non-tumor tissue samples (*n* = 30) are determined by qRT-PCR. **D** ZSCAN18 expression score analysis of IHC staining in GC tissues and adjacent non-tumor tissues (*n* = 83). Score of (−) or (+) is classified as negative, while score of (++) or (+++) is classified as positive (see above). **E** Kaplan–Meier curves show the association of overall survival rate of GC patients with ZSCAN18 expression. Green: GC patients with negative expression (*n* = 49, median survival, 21 months); orange: GC patients with positive expression (*n* = 34, median survival, 37 months, *p* < 0.001, log-rank test). **F** Multivariate analysis shows independent prognosis factors. **G** Bioinformatics analysis from the public datasets (GSE30601) in adjacent non-tumor tissues and tumor tissues. **H** Methylation status and levels of ZSCAN18 in GC tissue (*n* = 97) and adjacent non-tumor tissue (*n* = 6) samples are determined by MassARRAY. **I** The methylation levels of ZSCAN18 were detected by MassARRAY in GC tissues and adjacent non-tumor tissue samples, the CpG11.19.39, CpG23.37.44, CpG26.27 and CpG45.46 are significantly different. **J** Kaplan–Meier curves show the association of overall survival rate of GC patients with ZSCAN18 hypermethylation. **K** Multivariate analysis shows independent prognosis factors. Each experiment was performed in triplicate and repeated at least three times. Data were represented as mean ± SD. *p < 0.05, **p < 0.01, ***p < 0.001

Initially, bioinformatics analysis of the data from The Cancer Genome Atlas (TCGA) datasets showed that expression of ZSCAN18 was depressed in a variety of cancers (Fig. 1A), and analysis from the public datasets (GSE33335) showed that ZSCAN18 is downregulated in GC samples (Fig. 1B). Subsequently, we compared the mRNA expression of ZSCAN18 in 30 GC tissue samples and matched adjacent non-tumor tissue samples by qRT-PCR. Accordingly, ZSCAN18 was downregulated significantly in the primary tumor compared to that in the adjacent non-tumor tissues (Fig. 1C). Next, using IHC analysis, we semi-quantified ZSCAN18 protein levels of 83 fresh gastric adenocarcinoma tissues and matched adjacent non-tumor tissues. IHC demonstrated that ZSCAN18 was positively stained in the nuclei of gastric mucosal cells and that its protein levels were considerably lower in GC tissues than in matched adjacent non-tumor tissues (Fig. 1D). Kaplan–Meier analysis showed that the overall survival of patients with negative expressions of ZSCAN18 was significantly shorter than the survival of patients with positive expressions (Fig. 1E). Tumor size, depth of tumor invasion (pT_3–4_ vs pT_1-2_), lymph node metastasis (positive vs negative) and low expression of ZSCAN18 predicted an increased risk of cancer-related death in univariate analysis (Table 1). Multivariate analysis revealed that depth of tumor invasion (pT_3–4_ vs pT_1-2_), lymph node metastasis (positive vs negative) and expression of ZSCAN18 were independent prognostic factors (Fig. 1F). These results demonstrate that ZSCAN18 is downregulated in primary GC and is associated with poor survival.

**Table 1:** Univariate Cox proportional hazard models for overall survival of gastric cancer patients (*n* = 83)

Predictor	Univariate analysis	
	HR (95% CI)	*p*
*Gender*		
Female versus Male	0.880 (0.516–1.499)	0.637
*Age (year)*		
< 65 versus ≥ 65	0.755 (0.458–1.242)	0.268
*Tumor size (cm)*		
> 4 versus ≤ 4	3.561 (2.045–6.201)	< 0.001***
*Lauren type*		
Intestinal versus Diffuse	1.234 (0.687–2.214)	0.482
*Tumor location*		
Middle 1/3 versus Up 1/3	0.663 (0.274–1.605)	0.363
Low 1/3 versus Up 1/3	1.228 (0.586–2.571)	0.587
> 2/3 stomach versus Up 1/3	1.262 (0.585–2.720)	0.553
*Tumor invasion*		
pT_3-4_ versus pT_1-2_	4.976 (2.473–10.014)	< 0.001***
*Lymph node metastasis*		
Positive versus Negative	2.714 (1.488–4.949)	0.001**
*Expression level of ZSCAN18*		
High versus Low	0.258 (0.141–0.471)	< 0.001***

**p < 0.01; ***p < 0.001

### ZSCAN18 is frequently hypermethylated in primary GC and is associated with poor survival

To explore the regulation mechanism of ZSCAN18 in GC, we focused on epigenetic alterations, especially DNA promoter methylation. Bioinformatics analysis of the data from the public dataset (GSE30601) confirmed a high methylation level of ZSCAN18 in GC (Fig. 1G). The MassARRAY analysis spanned the promoter region from − 209 to 280, including 50 CpG islands (of which 41 were detectable), and was used to evaluate the methylation level. The level of ZSCAN18 methylation was investigated in 97 primary GC tissues and 6 adjacent non-tumor tissues by MassARRAY analysis, which identified significantly higher methylation of ZSCAN18 in GC (Fig. 1H). Aberrant ZSCAN18 promoter hypermethylation in various CpG islands (CpG11.19.39, CpG23.37.44, CpG26.27 and CpG45.46) was detected more often in GC tissues than in adjacent non-tumor tissues (Fig. 1I). As shown in the Kaplan–Meier survival curves, patients with GC and high ZSCAN18 methylation had significantly shorter survival times compared with those with GC who had low ZSCAN18 methylation (Fig. 1J). Tumor size, depth of tumor invasion (pT_3–4_ vs pT_1-2_), lymph node metastasis (positive vs negative) and hypermethylation in CpG45.46 predicted an increased risk of cancer-related death in univariate analysis (Table 2). Additional multivariate analysis showed that ZSCAN18 promoter hypermethylation in CpG45.46, depth of tumor invasion (pT_3–4_ vs pT_1-2_) and lymph node metastasis (positive vs negative) were independent prognostic factors (Fig. 1K). Collectively, these results reveal that ZSCAN18 is frequently hypermethylated in primary GC and that hypermethylation contributes to poor survival in patients with GC; also, the methylation level of CpG45.46 may be an important marker of prognosis.

**Table 2:** Univariate Cox proportional hazard models for overall survival of gastric cancer patients (*n* = 97)

Predictor	Univariate analysis	
	HR (95% CI)	*p*
*Gender*		
Female versus Male	0.916 (0.599–1.401)	0.685
*Age (year)*		
≥ 60 versus < 60	0.986 (0.653–1.487)	0.946
*Tumor size (cm)*		
> 4 versus ≤ 4	1.402 (0.834–2.357)	0.203
*Lauren type*		
Diffuse versus Intestinal	1.079 (0.677–1.721)	0.749
Mix versus Intestinal	1.231 (0.498–3.044)	0.653
*Tumor location*		
Middle 1/3 versus Up 1/3	0.942 (0.531–1.673)	0.839
Low 1/3 versus Up 1/3	0.704 (0.406–1.221)	0.211
> 2/3 stomach versus Up 1/3	0.880 (0.413–1.873)	0.740
*Tumor invasion*		
pT_3-4_ versus pT_1-2_	2.388 (1.293–4.410)	0.005**
*Lymph node metastasis*		
Positive versus Negative	4.758 (2.603–8.698)	< 0.001***
*Methylation (high versus low)*		
CpG11	1.134 (0.722–1.783)	0.585
CpG23	0.852 (0.425–1.708)	0.652
CpG26.27	1.221 (0.532–2.803)	0.637
CpG45.46	2.060 (1.301–3.261)	0.002**

**p < 0.01; ***p < 0.001

### The silencing of ZSCAN18 mediated by promoter hypermethylation in GC cells

**Fig. 2:**
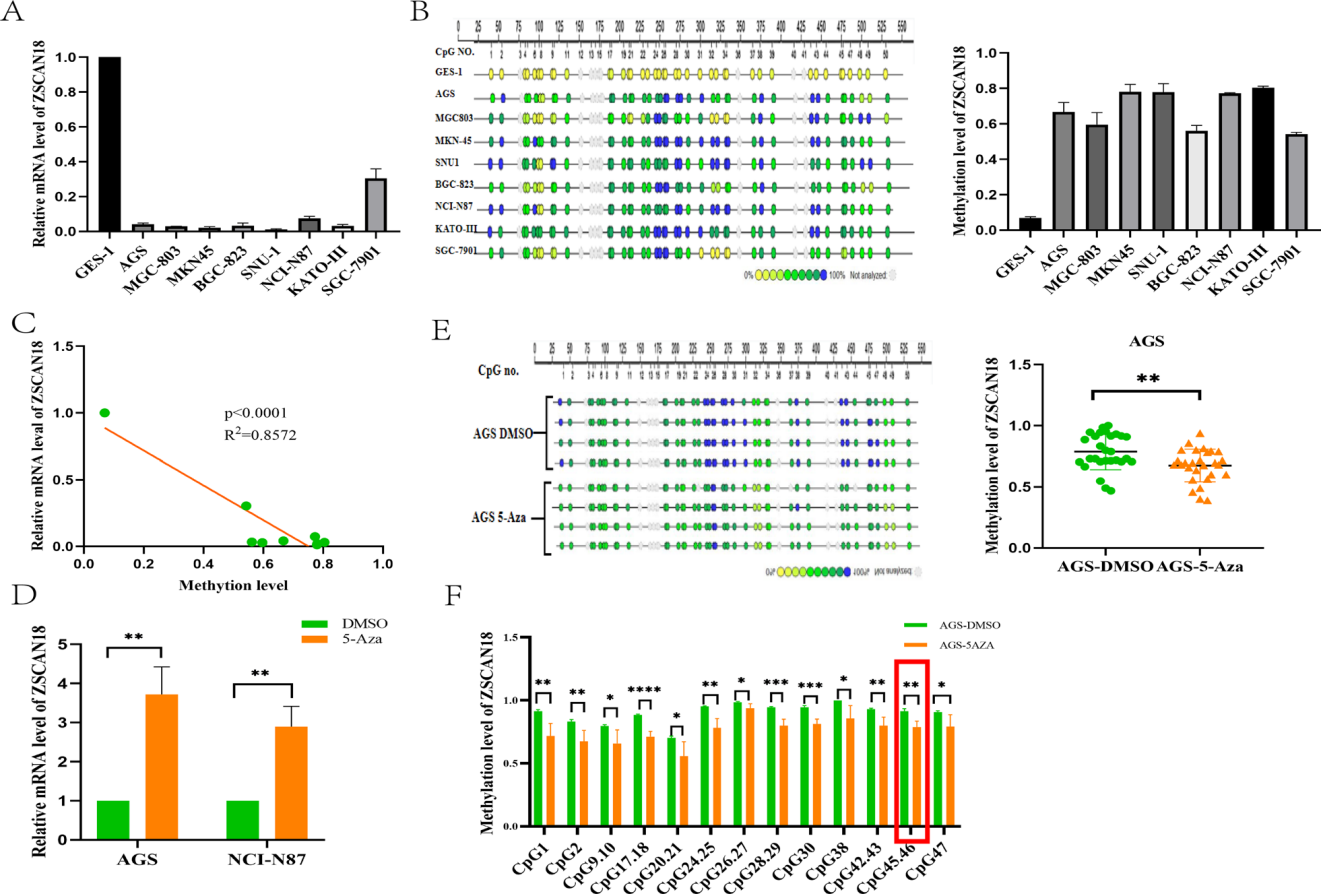
Demethylation upregulated ZSCAN18 expression in GC cell lines. **A** ZSCAN18 mRNA expression was reduced in all GC cell lines. GES-1, normal gastric epithelial cell line. **B** The methylation level of each GC cell line was significantly higher than that of GES-1. **C** Correlation between mRNA expression and methylation level of ZSCAN18 in GC cell lines. **D** The mRNA expression of ZSCAN18 in AGS and NCI-N87 cells was restored after treatment with demethylation agent 5-Aza-2′-deoxycytidine. **E** Demethylation of ZSCAN18 was observed in AGS cells after treatment with demethylation agent 5-Aza-2′-deoxycytidine as examined by MassARRAY analysis. **F** Methylation level of ZSCAN18 was detected in a variety of CpG islands by MassARRAY analysis. Each experiment was performed in triplicate and repeated at least three times. Data were represented as mean ± SD. *p < 0.05, **p < 0.01, ***p < 0.001, ****p < 0.0001

We examined the mRNA expression of ZSCAN18 in 8 GC cell lines by qRT-PCR. Reduced expression of ZSCAN18 was found in 8 GC cell lines compared with the human gastric epithelial cell line (GES-1) (Fig. 2A). We next explored the role of promoter methylation in the regulation of ZSCAN18 by MassARRAY analysis. Consistent with the reverse tendency of ZSCAN18 expression, hypermethylation was detected in these GC cell lines (Fig. 2B). A significant negative correlation was observed between mRNA expression and the methylation level of ZSCAN18 in the cell lines we detected (AGS, MGC-803, MKN45, SNU-1, BGC-823, NCI-N87, KATO-III and SGC-7901) (Fig. 2C). To confirm whether promoter methylation mediated ZSCAN18 silencing, two methylated cell lines (AGS and NCI-N87) that showed silencing of ZSCAN18 were treated with the DNA methyltransferase inhibitor DAC, and the mRNA expression of ZSCAN18 was restored in both lines (Fig. 2D). The change in the ZSCAN18 methylation level was synchronously validated by MassARRAY analysis in AGS cells (Fig. 2E). Furthermore, the decreased methylation level of ZSCAN18 was confirmed in a variety of CpG islands (Fig. 2F). These results indicate that promoter hypermethylation is associated with the transcriptional silence of ZSCAN18 in GC cells; moreover, additional demethylation restores the expression of ZSCAN18.

### Rescue of ZSCAN18 expression inhibits the proliferation of GC cells via triggering autophagy

**Fig. 3:**
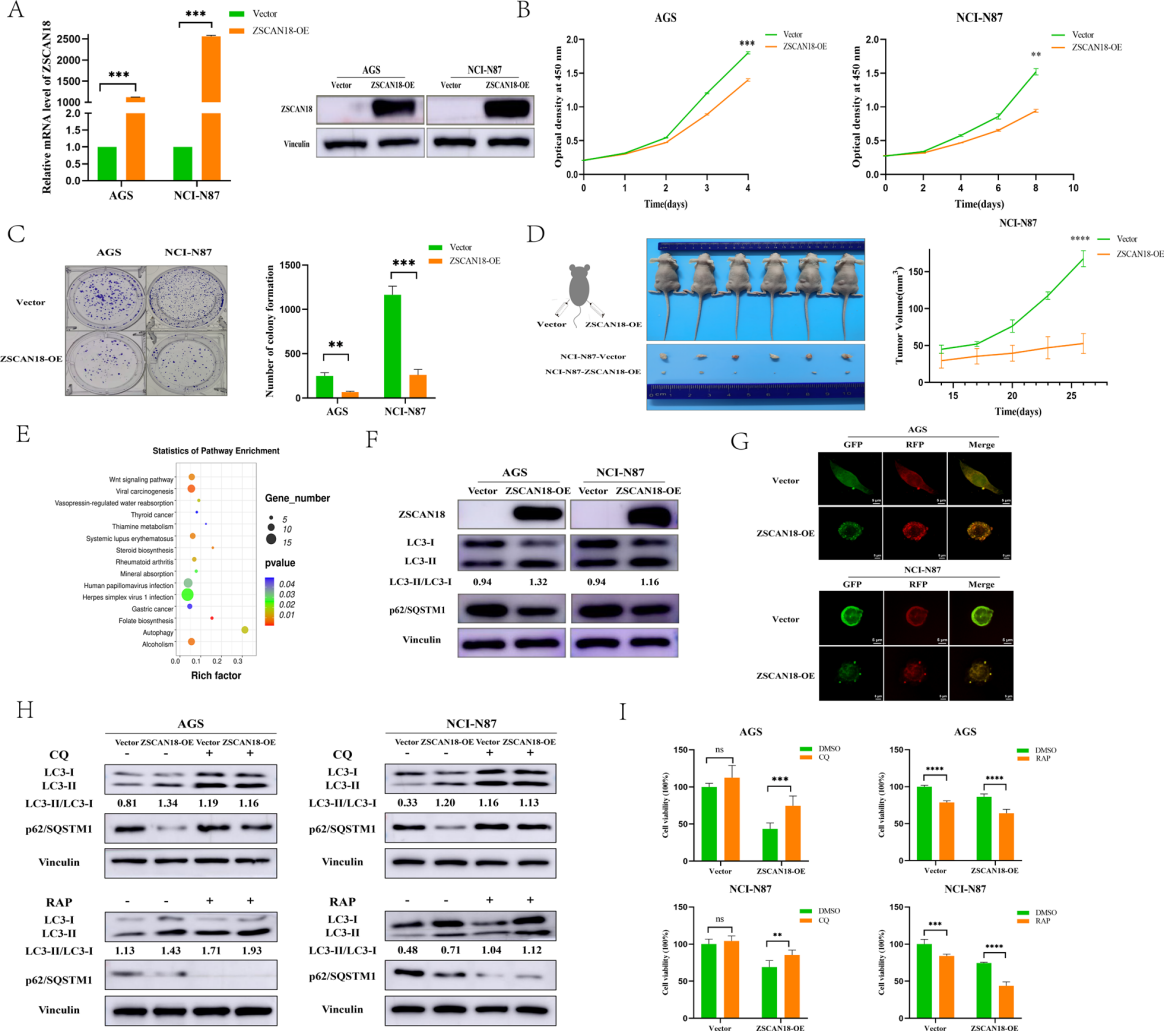
Rescue of ZSCAN18 expression inhibits the proliferation of GC cells via triggering autophagy. **A** Overexpression of ZSCAN18 in AGS and NCI-N87 cells was evidenced by qRT-PCR (left) and western blot (right). **B** Growth curves representing cell viability were inhibited by ZSCAN18 in both AGS and NCI-N87 cells. **C** Overexpression of ZSCAN18 suppressed colony formation in both AGS and NCI-N87 cells. **D** Tumor growth was inhibited in nude mice subcutaneously inoculated NCI-N87 cells with ZSCAN18 overexpression compared with control ones. **E** The KEGG enrichment plots of DEGs were generated from mRNA sequencing analysis of ZSCAN18-overexpressed and control NCI-N87 cells. **F** The expression of typical autophagy-related proteins was detected by western blot in ZSCAN18-overexpressed AGS and ZSCAN18-overexpressed NCI-N87 cells. **G** Immunofluorescent micrographs showed autophagosomes in both AGS and NCI-N87 cells. **H** The expression of typical autophagy-related proteins was detected by western blot in ZSCAN18-overexpressed AGS and ZSCAN18-overexpressed NCI-N87 cells pretreated with CQ/RAP. **I** The cell viability was detected by ATP in ZSCAN18-overexpressed AGS and ZSCAN18-overexpressed NCI-N87 cells after co-incubated with CQ/RAP. Each experiment was performed in triplicate and repeated at least three times. Data were represented as mean ± SD. **p < 0.01, ***p < 0.001, ****p < 0.0001

To elucidate the functional significance of ZSCAN18 in GC, we first upregulated ZSCAN18 in AGS and NCI-N87 cells, and overexpression of ZSCAN18 was identified by qRT-PCR and western blot (Fig. 3A). The CCK-8 assay showed that ZSCAN18 overexpression could inhibit the proliferation of both AGS and NCI-N87 cells (Fig. 3B). This inhibitory effect on cell growth was also confirmed with a colony formation assay in both AGS and NCI-N87 cells (Fig. 3C). Subsequently, NCI-N87 cell lines transfected with ZSCAN18 overexpression or empty vector control cells were used to establish xenograft tumors in mice to examine whether ZSCAN18 could suppress the growth of GC cells in vivo. The tumor volumes were 52.67 ± 13.58 mm^3^ in xenografted mice with ZSCAN18-overexpressed NCI-N87 cells and 167.7 ± 10.89 mm^3^ in xenografted mice with empty vector control NCI-N87 cells (Fig. 3D). The tumor growth was significantly lower in ZSCAN18-transfected nude mice than in the vector control mice, suggesting that ZSCAN18 does function as a tumor suppressor in GC. To gain insights into the molecular mechanisms underlying the tumor inhibition of ZSCAN18, we analyzed the downstream signaling pathways modulated by ZSCAN18 through RNA next-generation sequencing (RNA-seq) in ZSCAN18-overexpressed and control NCI-N87 cells. These DEGs were classified into different KEGG enrichment pathways, including the autophagy pathway, which was well established as playing an important role in carcinogenesis (Fig. 3E). To investigate the role of ZSCAN18 in the promotion of GC cells autophagy, autophagy‐related protein expression was assayed via western blotting. We observed increased LC3-II/LC3-I expression and decreased p62/SQSTM1 expression in AGS and NCI-N87 cells transfected with the ZSCAN18 overexpressing plasmid compared with the empty vector (Fig. 3F). In addition, we detected autophagic vesicles via fluorescence microscope. The number of yellow puncta increased significantly in the ZSCAN18 overexpression group, which confirmed the increase in the number of autophagosomes (Fig. 3G). To further illuminate the role of ZSCAN18-induced autophagy on cell proliferation, ZSCAN18-overexpressed AGS and ZSCAN18-overexpressed NCI-N87 cells were pretreated with CQ and RAP, respectively. We observed increased LC3-II/LC3-I expression and decreased p62/SQSTM1 expression in ZSCAN18-overexpressed AGS and ZSCAN18-overexpressed NCI-N87 cells pretreated with RAP compared with the empty vector and reversed results in p62/SQSTM1 expression by the addition of CQ (Fig. 3H). When co-incubated with CQ, the cell viability was significantly enhanced in ZSCAN18-overexpressed AGS and ZSCAN18-overexpressed NCI-N87 cells (Fig. 3I). Conversely, the cell viability was significantly inhibited in ZSCAN18-overexpressed AGS and ZSCAN18-overexpressed NCI-N87 cells when co-incubated with RAP (Fig. 3I). Above all, these findings demonstrated that the proliferation was inhibited, while the autophagy was enhanced in ZSCAN18 overexpression AGS and NCI-N87 cells indicating a modulation effect between ZSCAN18-induced autophagy and cell proliferation.

### TP53INP2 is transcriptionally regulated by ZSCAN18 in GC cell lines

**Fig. 4:**
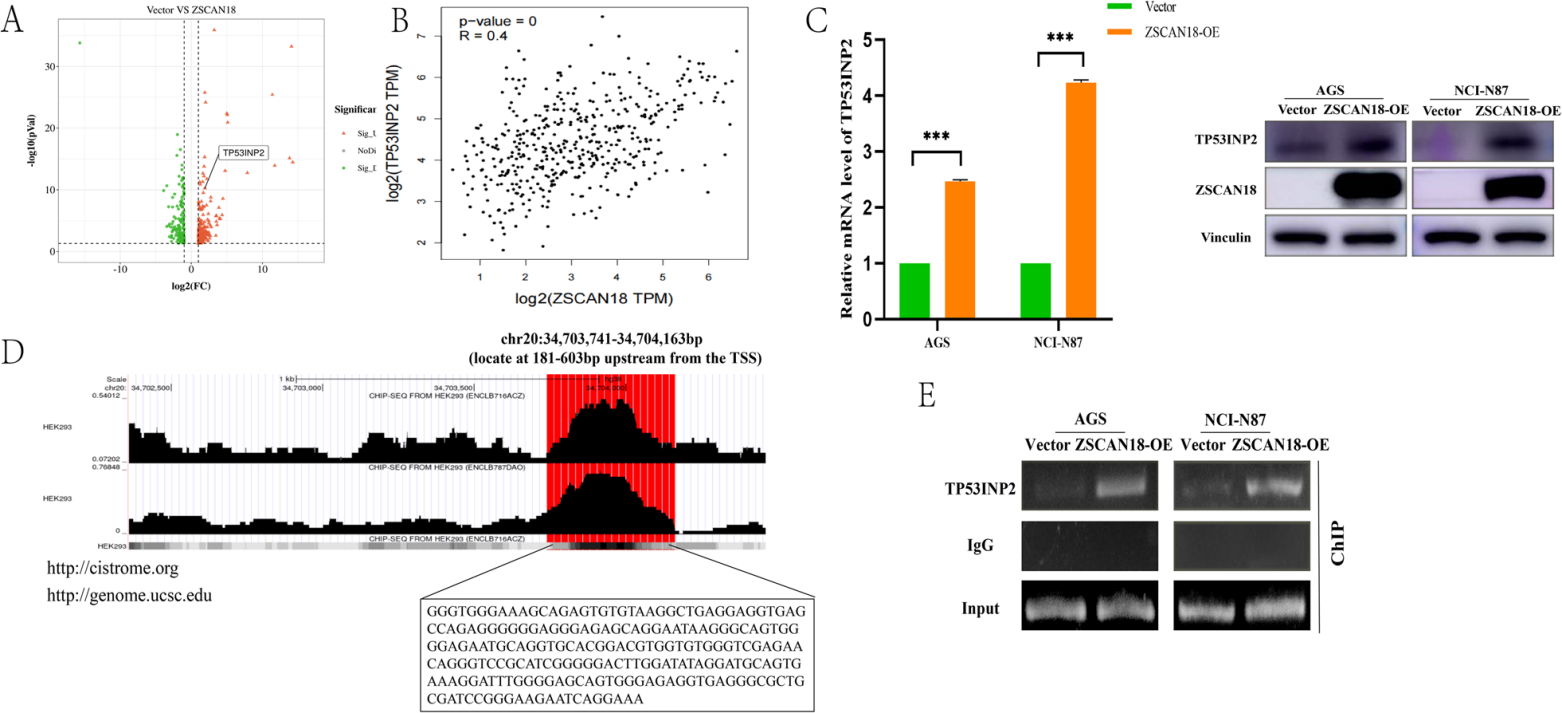
ZSCAN18 regulates TP53INP2 transcriptionally and binds to the TP53INP2 promoter region. **A** The volcano plots of DEGs were generated from mRNA sequencing analysis of ZSCAN18-overexpressed and control NCI-N87 cells. **B** ZSCAN18 is correlated with TP53INP2 in GC (http://gepia.cancer-pku.cn). **C** TP53INP2 increased after ZSCAN18 overexpression in AGS and NCI-N87 cells measured by qRT-PCR (left) and western blot (right). **D** ZSCAN18 was enriched in the promoter region of TP53INP2 according to bioinformation analysis (http://genome.ucsc.edu/cgi-bin). **E** Chip assay confirmed that ZSCAN18 could bind to the TP53INP2 promoter region in both AGS and NCI-N87 cells. Each experiment was performed in triplicate and repeated at least three times. Data were represented as mean ± SD. ***p < 0.001

As IHC results showed, ZSCAN18 localized mainly to the nucleus; thus, it is reasonable to speculate that ZSCAN18 might be implicated in the regulation of gene expression. We found that the expressions of 510 genes (DEGs) were significantly changed (FC > 2, *p* < 0.05) in NCI-N87 cells; among them, 289 genes were upregulated, whereas 221 were downregulated. Among these DEGs, TP53INP2 had attracted our attention because it is related to autophagy [12] (Fig. 4A). ZSCAN18 was positively correlated with TP53INP2 expression in GC tissues, according to bioinformatics analysis (Fig. 4B). The expression levels of TP53INP2 in AGS and NCI-N87 cells after ZSCAN18 overexpression were detected by qRT-PCR and western blot (Fig. 4C). ChIP-seq analysis according to bioinformatics analysis showed that ZSCAN18 enrichment was found in the promoter region of TP53INP2 (Fig. 4D). ChIP assay further confirmed that ZSCAN18 overexpression could bind more to the TP53INP2 promoter region than the empty vector (Fig. 4E). Taken together, these data suggest that TP53INP2 was transcriptional regulated by ZSCAN18.

### TP53INP2 attenuated the GC cell proliferation through inducing autophagy

**Fig. 5:**
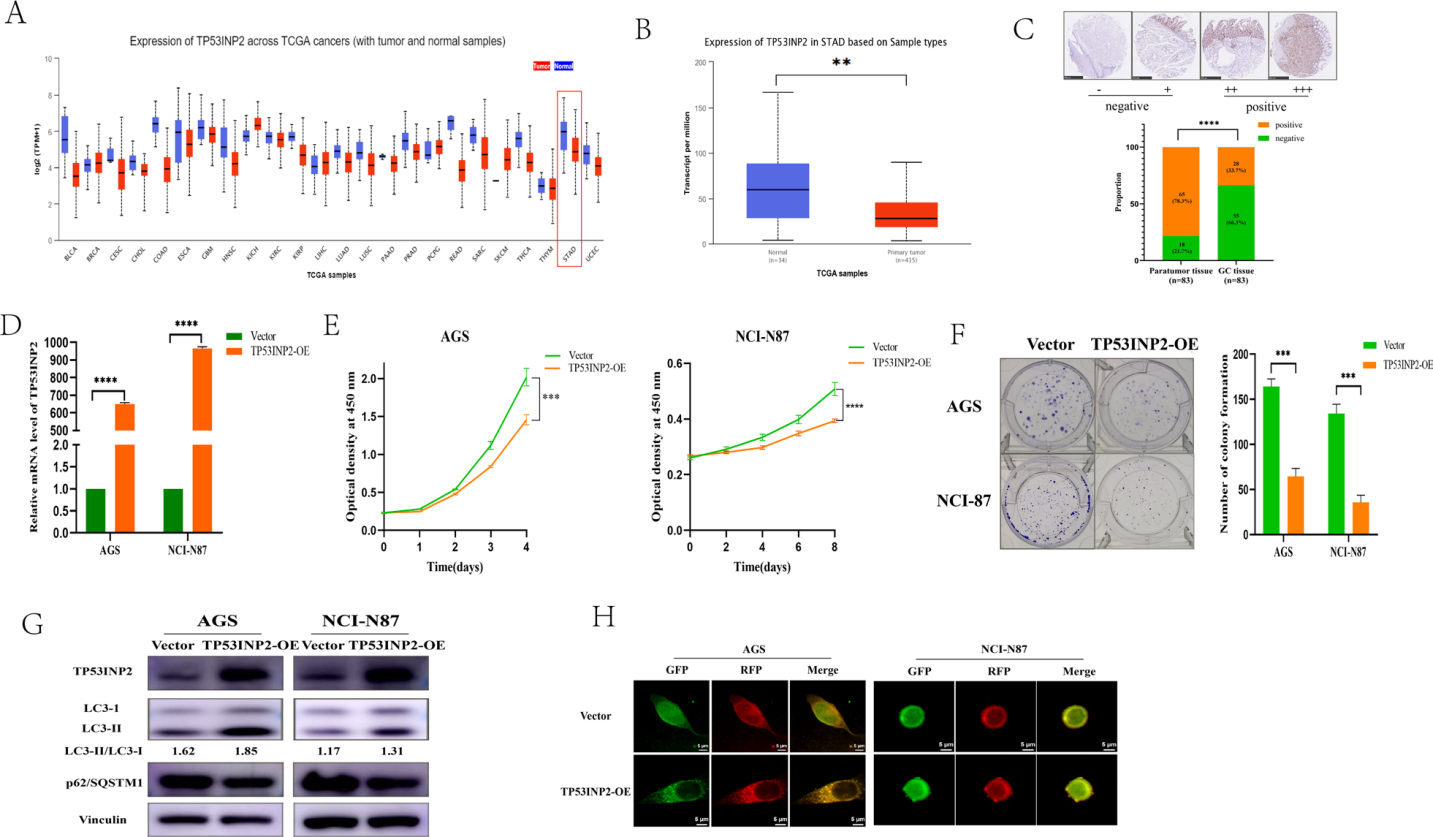
The effect of TP53INP2 overexpression on proliferation and autophagy of GC cells. **A** The expression of TP53INP2 in different types of carcinomas (http://ualcan.path.uab.edu). BLCA: bladder urothelial carcinoma; BRCA: breast invasive carcinoma; CESC: cervical squamous cell carcinoma and endocervical adenocarcinoma; CHOL: cholangiocarcinoma; COAD: colon adenocarcinoma; ESCA: esophageal carcinoma; GBM: glioblastoma multiforme; HNSC: head and neck squamous cell carcinoma; KICH: kidney chromophobe; KIRC: kidney renal clear cell carcinoma; KIRP: kidney renal papillary cell carcinoma; LIHC: liver hepatocellular carcinoma; LUAD: lung adenocarcinoma; LUSC: lung squamous cell carcinoma; PAAD: pancreatic adenocarcinoma; PRAD: prostate adenocarcinoma; PCPG: prostate adenocarcinoma; READ: rectum adenocarcinoma; SARC: sarcoma; SKCM: skin cutaneous melanoma; THCA: thyroid carcinoma; THYM: thymoma; STAD: stomach adenocarcinoma; UCEC: uterine corpus endometrial carcinoma. **B** The relative expression of TP53INP2 in GC and adjacent tissues from TCGA database (normal mucosa = 34, tumor tissue = 415). **C** IHC staining reveals TP53INP2 levels in GC tissue and para-carcinoma tissue. **D** Overexpression of TP53INP2 in AGS and NCI-N87 cells was evidenced by qRT-PCR. **E** Growth curves representing cell viability were inhibited by TP53INP2 in both AGS and NCI-N87 cells. **F** Overexpression of TP53INP2 suppressed colony formation in both AGS and NCI-N87 cells. **G** Western blotting analysis of TP53INP2, p62/SQSTM1 and LC3 in TP53INP2 overexpressed and control groups. **H** After stably transfected with tandem-labeled mRFP-GFP-LC3, representative images of mRFP-GFP-LC3 vector were shown by immunofluorescent detection. Each experiment was performed in triplicate and repeated at least three times. Data were represented as mean ± SD. **p < 0.01, ***p < 0.001, ****p < 0.0001

In most of the carcinomas, including GC, the expression of TP53INP2 was lower than in adjacent tissues, as bioinformatics analysis of the TCGA public datasets indicated (Fig. 5A, B) (http://ualcan.path.uab.edu). We examined the expression level of TP53INP2 in primary GC samples by IHC staining, which showed that the signal expression intensity of TP53INP2 in GC tissues was significantly lower than that in adjacent normal gastric tissues (Fig. 5C). To explore the biological functions of TP53INP2, TP53INP2 was overexpressed by transfection with TP53INP2 overexpression plasmid (Fig. 5D). Overexpression of TP53INP2 resulted in significantly inhibited cell growth (Fig. 5E) as well as reduced colony formation (Fig. 5F). TP53INP2 could induce autophagy by downregulating p62/SQSTM1, upregulating LC3‐II/LC3-I (Fig. 5G) and reducing the number of autophagosomes (Fig. 5H). These data indicate that TP53INP2 suppresses GC via inhibiting cell proliferation and inducing autophagy.

### ZSCAN18-induced suppression of GC cell proliferation is mediated by TP53INP2

**Fig. 6:**
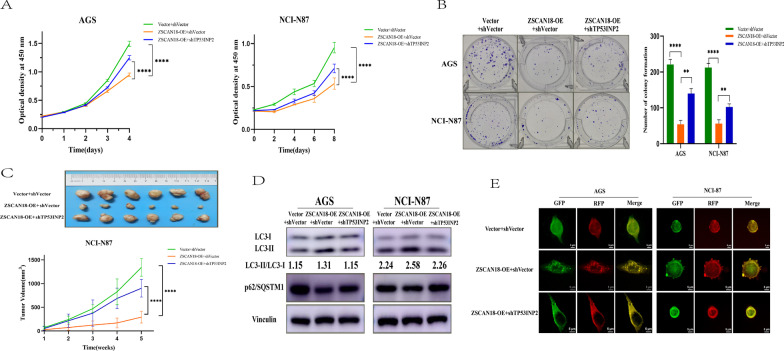
ZSCAN18-induced suppression of GC cell proliferation is mediated by TP53INP2. Three groups of cells were established in both AGS and NCI-N87 cells: ZSCAN18 overexpression and TP53INP2 knockdown (ZSCAN18 + shTP53INP2), ZSCAN18 overexpression and empty vector knockdown (ZSCAN18 + shVector), empty vector and empty vector knockdown (Vector + shVector). **A** Alteration of cell viability was detected among three groups in both AGS and NCI-N87 cells. **B** Alteration of colony formation was detected among three groups in both AGS and NCI-N87 cells. **C** The lethal effect in vivo was detected among three groups in NCI-N87 cells. **D**, **E** The autophagic flux was detected among three groups in both AGS and NCI-N87 cells. Each experiment was performed in triplicate and repeated at least three times. Data were represented as mean ± SD. ****p < 0.0001

Functional recovery experiments were performed to determine whether ZSCAN18 and TP53INP2 had a synergistic effect in GC. Three groups of cells were established in AGS and NCI-N87 cells: ZSCAN18 overexpression and TP53INP2 knockdown (ZSCAN18 + shTP53INP2), ZSCAN18 overexpression and empty vector (ZSCAN18 + shVector), empty vector and empty vector (Vector + shVector). Compared with the group of ZSCAN18 + shVector, the malignant progression of AGS and NCI-N87 cells in ZSCAN18 + shTP53INP2 group was significantly enhanced, which was characterized by promoted proliferation in vitro and in vivo (Fig. 6A–C), and impeded autophagy (Fig. 6D, E). In brief, the loss-/gain-of-function assays demonstrated that the knockdown of TP53INP2 could alleviate the inhibiting effects of ZSCAN18 overexpression in AGS and NCI-N87 cells.

## Discussion

In this study, we provide substantial evidence of a suppressive effect of ZSCAN18 on GC. We found that ZSCAN18 is widely downregulated in GC tissues and in most GC cell lines, suggesting a suppressive effect on carcinogenesis. As confirmed by demethylation treatment and MassARRAY analyses, these decreased expressions are mostly attributed to ZSCAN18 promoter hypermethylation in various CpG islands. However, ZSCAN18 hypermethylation was not detected in normal gastric mucosal tissues or GES-1 cells, suggesting that the hypermethylation of the ZSCAN18 promoter appears to follow a chronological sequence during carcinogenesis and is involved in the early stage of the multistep process of GC [9, 13, 14]. This study revealed that promoter hypermethylation is a unique mechanism of ZSCAN18 inactivation in GC. In addition, we confirmed for the first time, to our knowledge, that downregulation of ZSCAN18 with hypermethylation in CpG44.45 is associated with poor survival in primary GC; this finding suggests that ZSCAN18 may serve as a biomarker of poor prognosis [15–17]. Furthermore, the restorability of CpG44.45 after treatment with DAC (also known as decitabine, a chemotherapy agent) indicates that ZSCAN18 may be a potential target for an anti-tumor drug.

Given that ZSCAN18 reduction leads to poor survival, it is reasonable to postulate that ZSCAN18 could affect the progression and development of GC. Furthermore, the tumor-suppressive function of ZSCAN18 in GC was investigated both in vitro and in vivo. First, gain-of-function experiments using ZSCAN18 were performed in AGS and NCI-N87 cells, and the effect of ZSCAN18 on the proliferation of these cells was examined using CCK-8 and colony formation assays. The results showed that ZSCAN18 overexpression inhibited GC cell proliferation. In addition, decreased tumor growth resulting from ZSCAN18 overexpression was observed in nude mice and supports the inhibitory role of ZSCAN18 in GC. Taken together, these results confirm the crucial role of ZSCAN18 as a functional anti-tumor gene through suppression of cell proliferation in GC.

To extend our investigation of molecular mechanisms, we analyzed downstream signaling pathways regulated by ZSCAN18 through RNA-seq due to its role as a transcription factor. We elucidated the molecular mechanism of the tumor-suppressive function of ZSCAN18: regulation of the autophagy signaling pathway. In solid tumors, although autophagy has emerged as an attractive therapeutic target, the role of autophagy in tumor cells is complex and depends on tumor type and stage [18–21]. In this study, when ZSCAN18 is overexpressed, we find that autophagy is constitutively activated in AGS and NCI-N87 cells through observation on the elevated conversion of LC3-I to II, decreased expression of p62/SQSTM1, and increased autophagosomes. The proliferation modulated by ZSCAN18 in AGS and NCI-N87 cells is further confirmed by co-incubated with CQ (known as an autophagy inhibitor) and RAP (known as an autophagy activator), respectively.

Our experiment confirmed that abnormal overexpression of TP53INP2 caused by the ZSCAN18 upregulation plays an extremely important role in the suppression of GC. Moreover, TP53INP2 is a key positive regulator of autophagy and may act as a novel autophagic adaptor through the recruitment of ubiquitinated substrates to autophagosomes for degradation [22–25]. In our study, we confirmed that overexpression of TP53INP2 can enhance autophagy in GC cells.

Based on our data, we speculate that there may be a link between ZSCAN18 and TP53INP2. As a transcription factor, ZSCAN18 may transcriptively regulate TP53INP2 and bind to the promoter region of TP53INP2 to accelerate transcription and increased its protein expression. In addition, ChIP assay further confirmed that overexpression of ZSCAN18 could bind to the TP53INP2 promoter region and showed an interaction between ZSCAN18 and TP53INP2. We further confirmed the biological effects of ZSCAN18 regulation on TP53INP2 by in vitro and in vivo loss-/gain-of-function assays. Together, these data demonstrate that ZSCAN18 inhibits the malignant behavior of GC cells by binding to the TP53INP2 promoter.

Overall, the low expression of ZSCAN18 in GC tissues and cells is correlated with hypermethylation in the promoter region, which has clinical value in predicting the prognosis of GC. ZSCAN18 inhibits malignant behavior in GC by binding to the TP53INP2 promoter region and promoting autophagy, which may contribute to the development of a promising target for carcinoma diagnosis and treatment.

## Data Availability

The datasets used and/or analyzed during the current study are available from the corresponding author upon reasonable request.

## Abbreviations

GC: Gastric cancer
ZNF: Zinc finger
TF: Transcription factors
ZSCAN18: Zinc finger and scan domain containing 18
TP53INP2: Tumor protein 53-induced nuclear protein 2
GEO: Gene Expression Omnibus
IHC: Immunohistochemistry
FBS: Fetal bovine serum
DMEM: Dulbecco’s modified Eagle medium
qRT-PCR: Quantitative real-time polymerase chain reaction
CCK-8: Cell Counting Kit 8
ATP: Adenosine triphosphate
CQ: Chloroquine
RAP: Rapamycin
V: Volume
DAC: Decitabine/5-aza-2′-deoxycytidine
FC: Fold change
DEGs: Differentially expressed genes
KEGG: Kyoto Encyclopedia of Genes and Genomes
dNTPs: Deoxyribonucleoside-5′-triphosphates
ChIP-seq: Chromatin immunoprecipitation next-generation sequencing
RefSeq: Reference sequence
ChIP: Chromatin immunoprecipitation
IgG: Immunoglobulin G
ROC: Receiver operating characteristic
TCGA: The Cancer Genome Atlas
GES-1: Human gastric epithelial cell line
RNA-seq: RNA next-generation sequencing
